# Supplementary material for: Critical Review of Tuberculosis Diagnosis in Children from Papua New Guinea Presenting to Health Facilities in the Torres Strait Islands, Australia
Source: Microorganisms. 2023 Dec 8;11(12):2947. doi: 10.3390/microorganisms11122947 (PMC10745913; doi:10.3390/microorganisms11122947)
Supplement: Supplementary file 1 [file microorganisms-11-02947-s001.zip › microorganisms-2682038-supplementary.pdf]

Article

Critical review of tuberculosis diagnosis in children from Papua New Guinea presenting to health facilities in the Torres Strait Islands, Australia

Supplementary Materials

TABLE 1. Keith Edwards Score for Diagnosis of Tuberculosis in Children

Feature	Score		
	0	1	3
Duration of illness (weeks)	< 2	2-4	> 4
Nutrition (% of weight for age)	> 80	60-80	< 60
Family history of tuberculosis	None	Reported by family	Proven sputum positive

Score for Other Features if Present

**Feature**	**Score**
Unexplained fever, night sweats, no response to malaria treatment	2
Positive tuberculin test	3
Lymph nodes: large, painless, firm, soft sinus in neck/axilla	3
Malnutrition, not improving after 4 weeks	3
Central nervous system : change in temperament, fits with or without abnormal cerebrospinal fluid findings	3
Joint swelling, bone swelling, sinuses	3
Unexplained abdominal mass, ascites	3
Angle deformity of spine	4

A score of 7 or more is indicative of tuberculosis

Figure S1. Keith Edwards Score for Diagnosis of Tuberculosis in Children 1987[12]

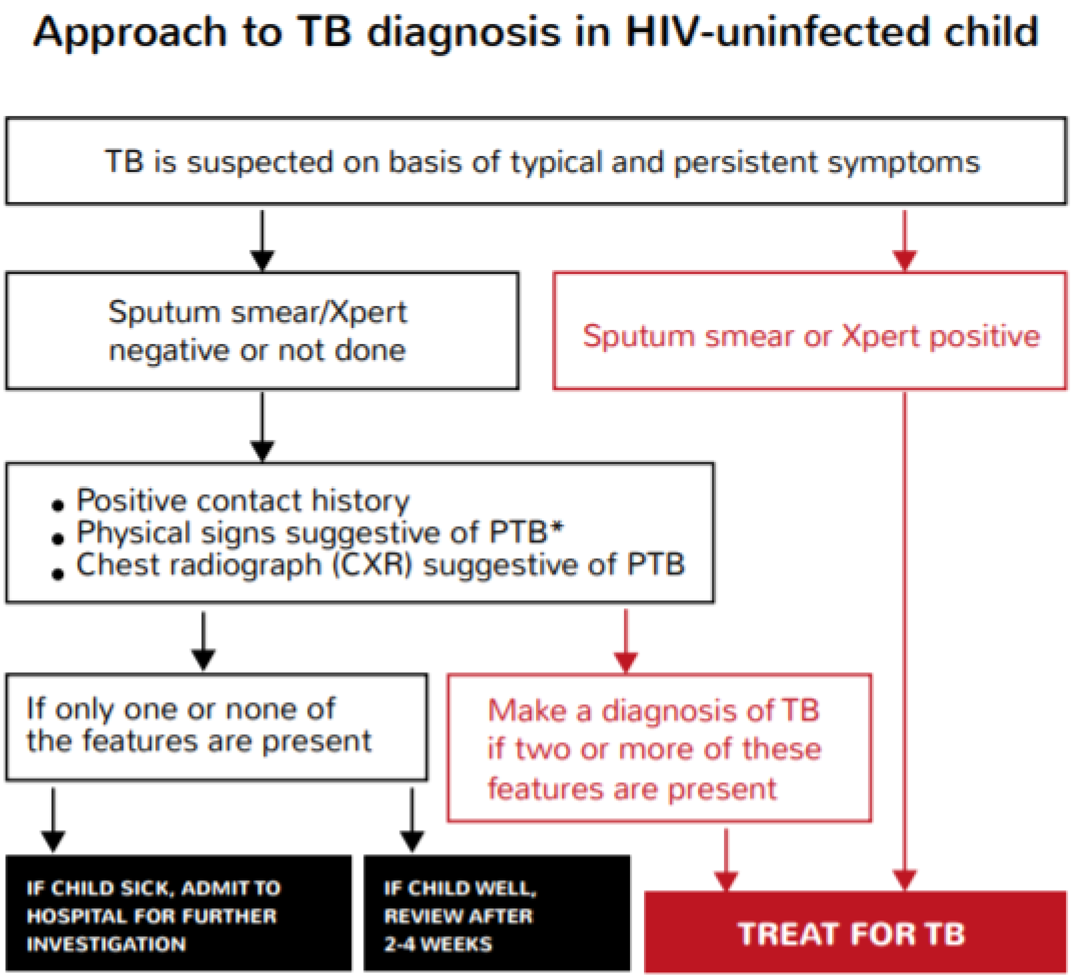

Figure S2. The Union Desk Guide 3rd Edition 2016[10]

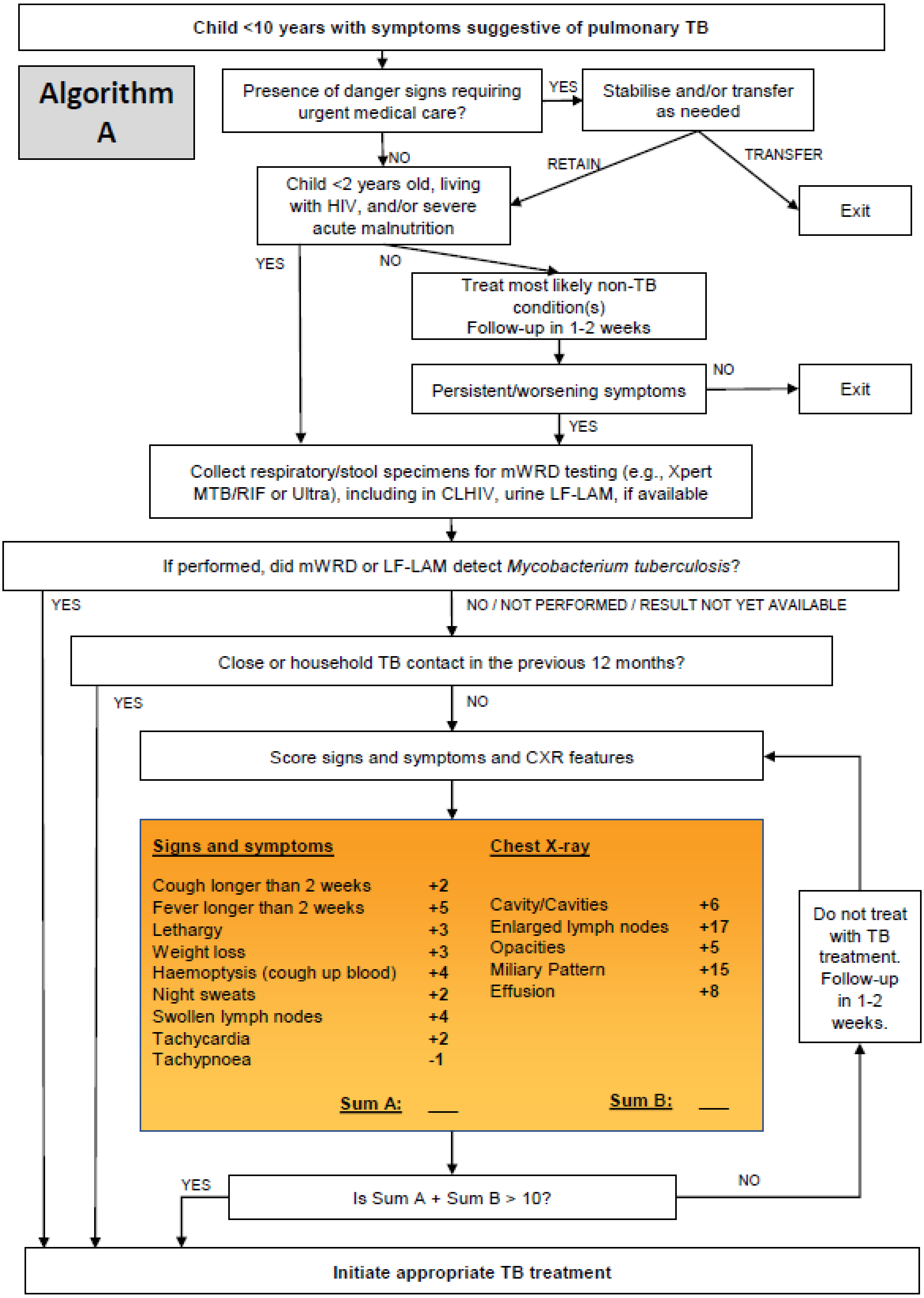

Figure S3. New World Health Organization Paediatric TB algorithm 2022[13]

Table S1. Description of established and modified scoring in Keith Edwards TB Score, The Union Desk Guide and the New WHO Algorithm

	KEITH EDWARDS TB SCORE (1987)	MODIFIED KEITH EDWARDS TB SCORE	UNION DESK GUIDE (2016)	MODIFIED UNION DESK GUIDE	NEW WHO ALGORITHM (2022)	MODIFIED WHO ALGORITHM
**DIAGNOSTIC FEATURE**	**SCORING SYSTEM (POINTS-BASED)**		**DIAGNOSTIC GUIDE**		**OPERATIONAL ALGORITHM**	
**COUGH**	0 - <2 weeks<br>1 – 2-4 weeks<br>3 - >4 weeks	0 - <2 weeks<br>1 – 2-4 weeks<br>3 - >4 weeks	0 – ≤2 weeks<br>1 – >2 weeks	0 – ≤2 weeks<br>1 – >2 weeks	0 – ≤2 weeks<br>2 – >2 weeks	0 – ≤2 weeks<br>2 – >2 weeks
**FEVER**	0 - <2 weeks<br>1 – 2-4 weeks<br>3 - >4 weeks	0 – ≤2 weeks<br>2 – >2 weeks	0 – ≤2 weeks<br>1 – >2 weeks	0 – ≤2 weeks<br>1 – >2 weeks	0 – ≤2 weeks<br>5 – >2 weeks	0 – ≤2 weeks<br>5 – >2 weeks
**WEIGHT LOSS**	3 - malnutrition not improving after four weeks of nutritional rehabilitation	Excluded **	0 – not reported<br>1 – reported	0 – not reported<br>1 – reported	0 – not reported<br>3 – reported	0 – not reported<br>3 – reported
**NIGHT SWEATS**	0 - <2 weeks<br>1 – 2-4 weeks<br>3 - >4 weeks	0 – ≤2 weeks<br>2 – >2 weeks	0 – ≤2 weeks<br>1 – >2 weeks	0 – ≤2 weeks<br>1 – >2 weeks	0 – not reported<br>2 – reported	0 – not reported<br>2 – reported
**SWOLLEN LYMPH NODES**	0 – not present<br>3 – present	0 – not present<br>3 – present	Not specified	0 – not present<br>1 – present	0 – not reported<br>4 – reported	0 – not reported<br>4 – reported
**TB CONTACT**	0 – no family history of TB	0 – no family history of TB	0 – no TB contact history	0 – no TB contact history	0 – no TB contact history in	0 – no TB contact history in previous 12 months

	KEITH EDWARDS TB SCORE (1987)	MODIFIED KEITH EDWARDS TB SCORE	UNION DESK GUIDE (2016)	MODIFIED UNION DESK GUIDE	NEW WHO ALGORITHM (2022)	MODIFIED WHO ALGORITHM
**DIAGNOSTIC FEATURE**	**SCORING SYSTEM (POINTS-BASED)**		**DIAGNOSTIC GUIDE**		**OPERATIONAL ALGORITHM**	
	1 – contact with sputum smear negative TB<br>3 – contact with sputum smear positive TB	3* – known TB contact of either sputum smear positive or negative	1 – TB contact history	1 – TB contact history	previous 12 months<br>0 – TB contact history in previous 12 months without persistent symptoms<br>Treat - TB contact history in previous 12 months with persistent symptoms	0 – TB contact history in previous 12 months without persistent symptoms<br><br>Treat - TB contact history in previous 12 months with persistent symptoms
**WEIGHT-FOR-AGE (WFA)**	0 - >80% expected WFA<br>1 - 60-80% WFA<br>3 - <60% WFA	0 - ≥15th WFA percentile<br>1 - 3-14th percentile<br>3 - < 3rd percentile	N/A	N/A	Severe Acute Malnutrition – eligible to progress to through until the end of the algorithm	< 3rd percentile - eligible to progress to through until the end of the algorithm

	**KEITH EDWARDS TB SCORE (1987)**	**MODIFIED KEITH EDWARDS TB SCORE**	**UNION DESK GUIDE (2016)**	**MODIFIED UNION DESK GUIDE**	**NEW WHO ALGORITHM (2022)**	**MODIFIED WHO ALGORITHM**
**DIAGNOSTIC FEATURE**	**SCORING SYSTEM (POINTS-BASED)**		**DIAGNOSTIC GUIDE**		**OPERATIONAL ALGORITHM**	
**HIV**	N/A	N/A	N/A***	N/A	HIV positive - eligible to progress to through until the end of the algorithm	HIV positive - eligible to progress to through until the end of the algorithm
**TB INFECTION**	3 – positive tuberculin skin test	Excluded	N/A	N/A	N/A	N/A
**CHEST X-RAY FINDINGS**	N/A	N/A	0 – Not suggestive of TB or nor done<br>1 – Suggestive of TB	0 – Not suggestive of TB or nor done<br>1 – Suggestive of TB	0 – Not suggestive<br>6 – Cavities<br>17 – Enlarged lymph nodes<br>5 – Opacities<br>15 – Miliary pattern<br>8 – Effusion	0 – Not suggestive<br>6 – Cavities<br>17 – Enlarged lymph nodes<br>5 – Opacities<br>15 – Miliary pattern<br>8 – Effusion
**SPECIMEN FOR SMEAR/XPERT**	N/A	N/A	0 – Sputum smear/ Xpert negative or not done	0 – Sputum smear/ Xpert/culture negative or not done	0 – Sputum smear/ Xpert negative or not done	0 – Xpert/culture negative or not done<br>Treat - Xpert/culture positive

	**KEITH EDWARDS TB SCORE (1987)**	**MODIFIED KEITH EDWARDS TB SCORE**	**UNION DESK GUIDE (2016)**	**MODIFIED UNION DESK GUIDE**	**NEW WHO ALGORITHM (2022)**	**MODIFIED WHO ALGORITHM**
**DIAGNOSTIC FEATURE**	**SCORING SYSTEM (POINTS-BASED)**		**DIAGNOSTIC GUIDE**		**OPERATIONAL ALGORITHM**	
			1 – Sputum smear or Xpert positive	1 – Sputum smear/ Xpert/culture +	Treat – Sputum smear or Xpert positive	
**CNS**	3	3	N/A	N/A	N/A	N/A
**SKELETAL**	3	3	N/A	N/A	N/A	N/A
**ABDOMINAL**	3	3	N/A	N/A	N/A	N/A
**ANGLE DEFORMITY OF SPINE**	4	4	N/A	N/A	N/A	N/A
**SCORE INTERPRETATION**	Treat if score is ≥7		Treat if ≥2 features are present		Treat if score is >10	

CNS: Central nervous system: change in temperament, fits +/- abnormal cerebrospinal fluid findings

Skeletal: Joint swelling, bone swelling, sinuses

Abdominal: Unexplained abdominal mass, ascites
